# A Family of *Salmonella* Type III Secretion Effector Proteins Selectively Targets the NF-κB Signaling Pathway to Preserve Host Homeostasis

**DOI:** 10.1371/journal.ppat.1005484

**Published:** 2016-03-02

**Authors:** Hui Sun, Jana Kamanova, Maria Lara-Tejero, Jorge E. Galán

**Affiliations:** Department of Microbial Pathogenesis, Yale University School of Medicine, New Haven, Connecticut, United States of America; University of Toronto, CANADA

## Abstract

Microbial infections usually lead to host innate immune responses and inflammation. These responses most often limit pathogen replication although they can also result in host-tissue damage. The enteropathogenic bacteria *Salmonella* Typhimurium utilizes a type III secretion system to induce intestinal inflammation by delivering specific effector proteins that stimulate signal transduction pathways resulting in the production of pro-inflammatory cytokines. We show here that a family of related *Salmonella* Typhimurium effector proteins PipA, GogA and GtgA redundantly target components of the NF-κB signaling pathway to inhibit transcriptional responses leading to inflammation. We show that these effector proteins are proteases that cleave both the RelA (p65) and RelB transcription factors but do not target p100 (NF-κB2) or p105 (NF-κB1). A *Salmonella* Typhimurium strain lacking these effectors showed increased ability to stimulate NF-κB and increased virulence in an animal model of infection. These results indicate that bacterial pathogens can evolve determinants to preserve host homeostasis and that those determinants can reduce the pathogen’s virulence.

## Author Summary

The inflammatory response to microbial pathogens usually limits their replication but it can also cause tissue damage. The enteropathogenic bacteria *Salmonella* Typhimurium stimulate host signal transduction pathways that result in inflammation. We show here that a family of related *Salmonella* Typhimurium effector proteins, PipA, GogA and GtgA, which are delivered by its type III secretion systems, specifically and redundantly target components of the NF-κB signaling pathway to inhibit transcriptional responses leading to host inflammation. We show that these effector proteins are proteases that cleave both the RelA (p65) and RelB transcription factors, which are central components of the NF-κB signaling pathway, but do not target p100 (NF-κB2) or p105 (NF-κB1). A *Salmonella* Typhimurium mutant strain lacking these effector proteins showed increased ability to stimulate NF-κB and increased virulence in an animal model of infection. These results indicate that bacterial pathogens can evolve determinants to preserve host homeostasis.

## Introduction

Bacterial pathogens that have sustained long-standing associations with their hosts have evolved complex adaptations to modulate host functions to ensure their survival and replication [1–3]. One of these adaptations is the type III protein secretion system (T3SS), a complex multi-protein machine that delivers bacterially-encoded proteins into host cells [4]. These bacterial proteins, known as effectors, have the capacity to modulate or interfere with a variety of cellular processes [5]. *Salmonella enterica* serovar Typhimurium (*S*. Typhimurium), a cause of severe gastroenteritis in humans, encodes two T3SSs within its pathogenicity island 1 (SPI-1) and 2 (SPI-2), which in a coordinated fashion deliver more than 40 effector proteins into target host cells [6–8]. These effectors mediate bacterial entry, survival, and replication within host cells. In addition, through its T3SSs, *Salmonella* stimulates signal transduction pathways leading to the activation of Nuclear Factor κB (NF-κB), Signal Transducer and Activator of Transcription 3 (STAT3), and Mitogen Activated Protein (MAP) kinase pathways, which result in pro-inflammatory cytokine production and host inflammation [9–11]. Although inflammation is usually viewed as a host defense response that limits pathogen replication, for *S*. Typhimurium the stimulation of inflammation is necessary to acquire essential nutrients and respiration substrates that become available only in inflamed intestinal tissues [12, 13]. The biochemical activities of several effector proteins delivered by the *S*. Typhimurium T3SSs are well characterized [6, 14–23]. However, the function of most effector proteins remains unknown. In this study, we have examined the function of PipA, GtgA, and GogA, three highly related TTSS effector proteins from *S*. Typhimurium. We have found that a *S*. Typhimurium strain lacking these effector proteins exhibits increased lethality in an animal model of infection. We found that these effectors are proteases that target NF-κB transcription factors thus preventing transcriptional responses leading to inflammation. These findings indicate that a bacterial pathogen can evolve determinants to preserve host homeostasis and that those determinants can actually reduce their virulence.

## Results

### Absence of PipA, GtgA, and GogA results in increased *S*. Typhimurium virulence in a mouse model of infection

**Fig 1:**
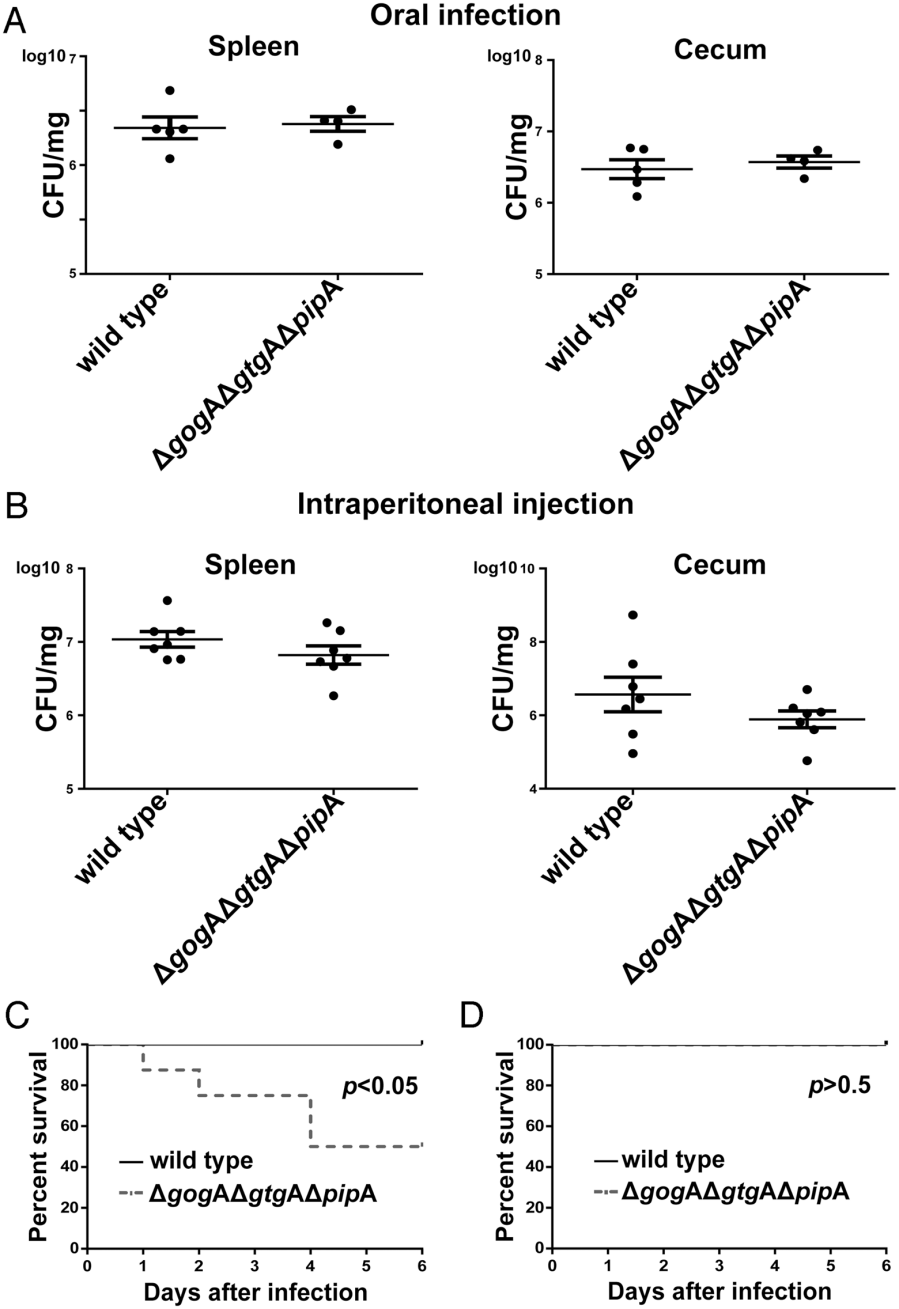
Absence of the PipA-family of effector proteins increases the mouse virulence of *S*. Typhimurium without increasing bacterial loads. (**A** and **B**) C57/BL6 *nramp* ^+/+^ mice were orally (**A**) or intraperitoneally (**B**) infected with wild-type S. *Typhimurium* or the *ΔpipA ΔgogA ΔgtgA* mutant and bacterial loads in the indicated tissues enumerated 7 days after infection. Each circle represents the bacterial load for an individual animal and horizontal bars indicate geometric means. The results are the combination of two independent experiments. (**C** and **D**) Survival of animals orally (**C**) or intraperitoneally (**D**) infected with wild-type *S*. Typhimurium (n = 7) or the *ΔpipA ΔgogA ΔgtgA* mutant (n = 8) strains 6 days after infection. The *p* values of the difference in the survival of animals infected with wild type or mutant strains determined by the log-rank test are shown. The results are the combination of two independent experiments.

PipA, GtgA, and GogA are three highly related *Salmonella* effector proteins (S1 Fig) encoded within the *Salmonella* pathogenicity island 5 (PipA) [24] or within lysogenic phages (GtgA and GogA) [25, 26]. These effectors are highly conserved in *Salmonella enterica* in that all known isolates encode at least one member of this family. In addition, homologs can also be detected in some pathogenic strains of *Escherichia coli* and the endosymbiont *Arsenophonus nasoniae* (S1 Fig) although no information is available about their potential function. Despite their widespread distribution within *Salmonellae*, nothing is known about their potential contribution to *Salmonella* virulence. In an effort to gain insight into the potential function of these effectors we constructed a *S*. Typhimurium strain simultaneously lacking these three effector proteins and examined its phenotype in a mouse model of infection. We inoculated orally or intraperitoneally otherwise identical mice (C57BL/6) expressing either wild type (resistant) or mutant (susceptible) alleles of NRAMP1 (SLC11A1), a divalent metal ion transporter that is known to significantly influence mouse-susceptibility to *S*. Typhimurium infection [27]. We found no significant differences in the levels of colony forming units (c. f. u.) of the wild type and *ΔgogA ΔgtgA ΔpipA S*. Typhimurium mutant strains in the different tissues of both mouse strains after oral or intraperitoneal infection (Fig 1A and 1B and S2 and S3 Figs). Surprisingly, however, despite the presence of equivalent bacterial burden we observed that a significant proportion of mice orally inoculated with the *S*. Typhimurium *ΔgogA ΔgtgA ΔpipA* mutant strains succumbed to infection earlier than animals inoculated with wild type (Fig 1C and S4A Fig). This difference was only apparent when animals were inoculated via the oral route (Fig 1C and 1D). Even more unexpected was the observation that this phenotype was apparent in mice that express a wild type allele of NRAMP1 (SLC11A1) (Fig 1C and S4A Fig) but not in mice expressing a mutant allele of this transporter and therefore more susceptible to wild-type *S*. Typhimurium (S4B Fig).

**Fig 2:**
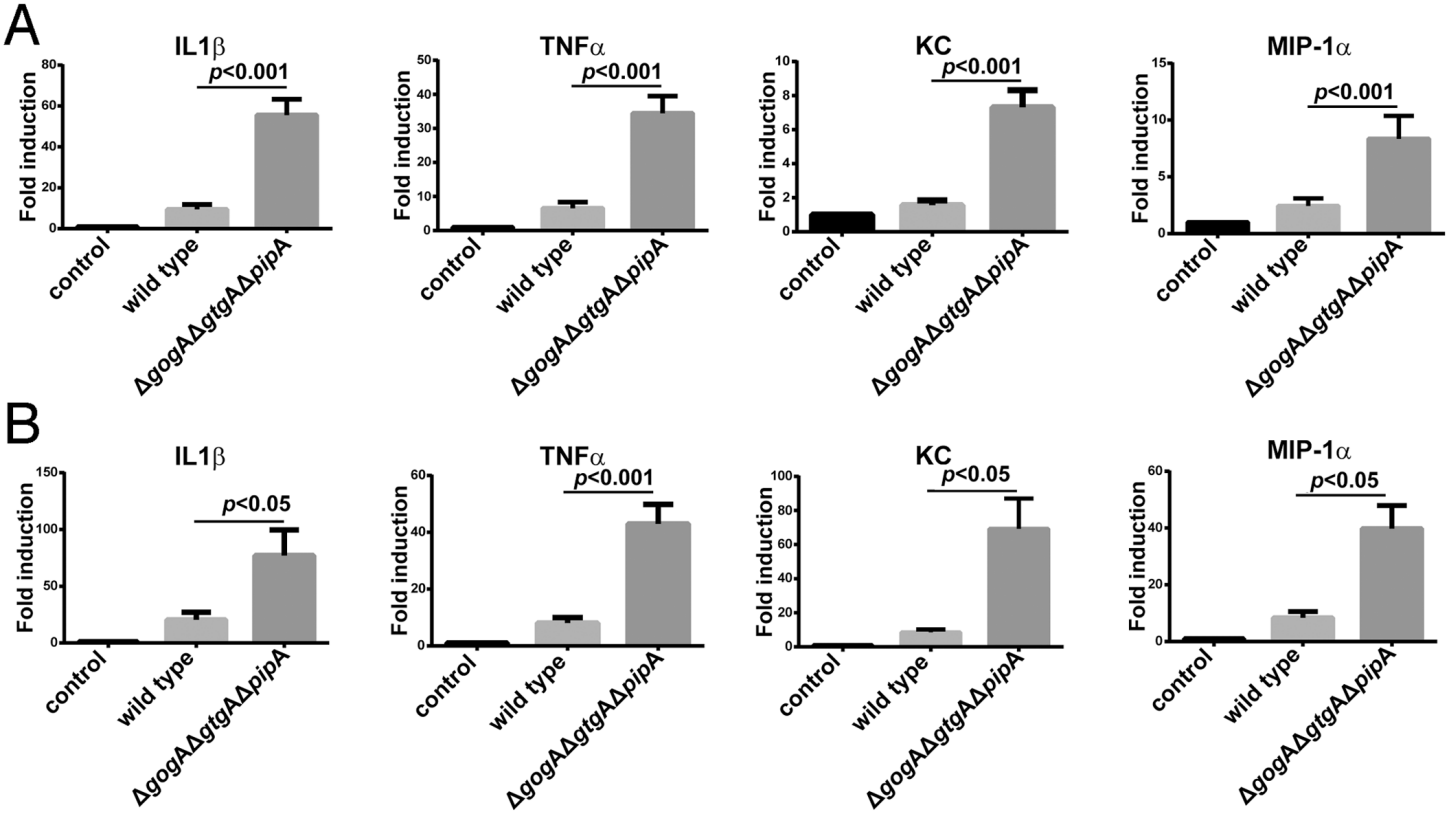
Absence of the PipA-family of effector proteins increases the ability of *S*. Typhimurium to stimulate pro-inflammatory cytokine expression in the mouse intestine. (**A** and **B**) C57/BL6 *nramp* ^+/+^ mice were orally infected with wild type (n = 5) or *ΔpipA ΔgogA ΔgtgA* (n = 5) *S*. Typhimurium strains and 24 (**A**) or 48 (**B**) hours after infection the relative levels of the indicated cytokines in the intestine were measured by quantitative PCR. Data were normalized to the levels of GAPDH and are expressed relative to uninfected control animals (n = 5). The data shown were compiled from two independent experiments of three measurements each. *p* values of the indicated differences determined by the Student *t* test are shown.

Since we observed equivalent number of bacterial loads in tissues infected with wild type or the mutant strain, we hypothesized that infection with the *S*. Typhimurium *ΔgogA ΔgtgA ΔpipA* mutant may result in increased production of pro-inflammatory cytokines in the intestinal epithelium, which may lead to increased lethality. Consistent with this hypothesis we observed higher levels of cytokines mRNA (Fig 2A and 2B) and more severe inflammation (S6 Fig) in the intestine of mice infected with the *S*. Typhimurium *ΔgogA ΔgtgA ΔpipA* mutant strain than in those infected with wild-type bacteria. We also measured the levels of a selected group of cytokines in the serum of infected animals and although animals infected with the *ΔgogA ΔgtgA ΔpipA* mutant strain exhibited higher levels of the measured cytokines than those infected with wild type, the differences did not approach statistical significance (S5 Fig). These results indicate that absence of GogA, GtgA, and PipA results in a more severe inflammatory response to *S*. Typhimurium, with increased cytokine production early in infection, which may be responsible for the heightened lethality.

### Infection of cultured cells with the *S*. Typhimurium *ΔgogA ΔgtgA ΔpipA* mutant strain results in increased NF-κB signaling

**Fig 3:**
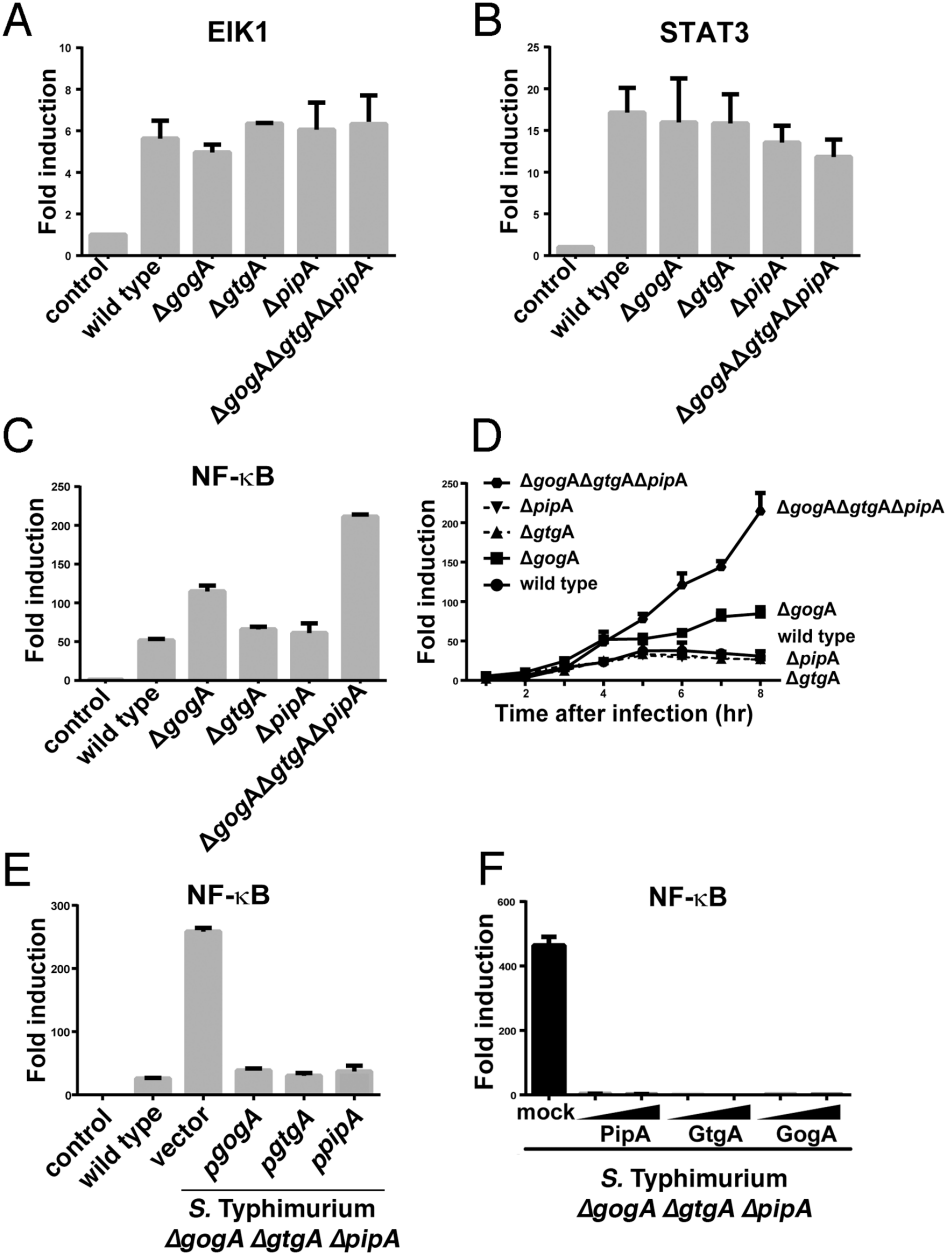
The PipA family of effector proteins negatively regulates *Salmonella*-induced NF-κB signaling. **(A-E**) HEK293T cells were transfected with plasmids encoding Elk1 (**A**), STAT3 (**B**) or NF-κB (**C-E**) signaling reporter constructs. Eighteen hours after transfection, cells were infected with the indicated *S*. Typhimurium strains at a MOI = 5 and the reporter activity was measured at 8 hours after infection (**A**-**C**, **E**) or at the indicated times after infection (**D**). Data are shown relative to the activity of the reporter in uninfected control cells and represent the mean ± standard deviation of three independent measurements. (**F**) HEK293T cells were transfected with a plasmid encoding a NF-κB reporter construct along with 25 or 50 ng of a plasmid encoding PipA, GtgA, or GogA. Eighteen hours after transfection, cells were infected with *S*. Typhimurium Δ*gog*A Δ*gtg*A Δ*pip*A at a MOI = 5 and the reporter activity was measured 8 hs after infection. Data are shown relative to the activity of the reporter in uninfected control cells and represent the mean ± standard deviation of three independent measurements.

In an effort to better understand the phenotype of the *S*. Typhimurium *ΔgogA ΔgtgA ΔpipA* mutant observed in experimental animals, we examined this mutant strain with several assays for phenotypes that previous studies have shown to be dependent on the function of the SPI-1- and/or SPI-2-encoded T3SSs [6–8]. We found that the *S*. Typhimurium *ΔgogA ΔgtgA ΔpipA* mutant strain exhibited wild type levels of invasion and replication within cultured cells (S7A and S7B Fig). Also through the activity of its T3SSs, *S*. Typhimurium stimulates a profound reprogramming of gene expression of the infected cells by stimulating mitogen activated protein (MAP) kinase, NF-κB and signal transducer and activator of transcription 3 (STAT3) signaling pathways [9–11, 28]. We found that the *S*. Typhimurium *ΔgogA ΔgtgA ΔpipA* mutant strain activated the MAP kinase-dependent transcription factor Elk1 and STAT-3 signaling in a manner indistinguishable from wild type (Fig 3A and 3B). In contrast, we found that this mutant strain activated the NF-κB signaling pathway significantly more robustly than wild type (Fig 3C). The enhanced activation of NF-κB in cells infected with the mutant strain was seen starting at 4 hs after infection and was more apparent later (8 hs) in infection (Fig 3D). In contrast *S*. Typhimurium strains carrying single deletion mutations in *pipA*, *gtgA*, or *gogA* showed little (*ΔgogA*) to no *(ΔgtgA* or *ΔpipA*) enhancement in their ability to activate NF-κB (Fig 3C and 3D). The phenotype of the *ΔgogA ΔgtgA ΔpipA* triple mutant could be complemented *in trans* by expressing plasmid-encoded *pipA*, *gtgA* or *gogA* (Fig 3E). Furthermore, transient expression of PipA, GtgA, or GogA in cultured mammalian cells completely abolished NF-κB activation by the *S*. Typhimurim *ΔgogA ΔgtgA ΔpipA* mutant strain (Fig 3F). These results indicate that these three effectors work in a redundant/dose-dependent fashion to inhibit *Salmonella*-induced NF-κB signaling.

### The PipA family of effectors can inhibit NF-κB signaling

**Fig 4:**
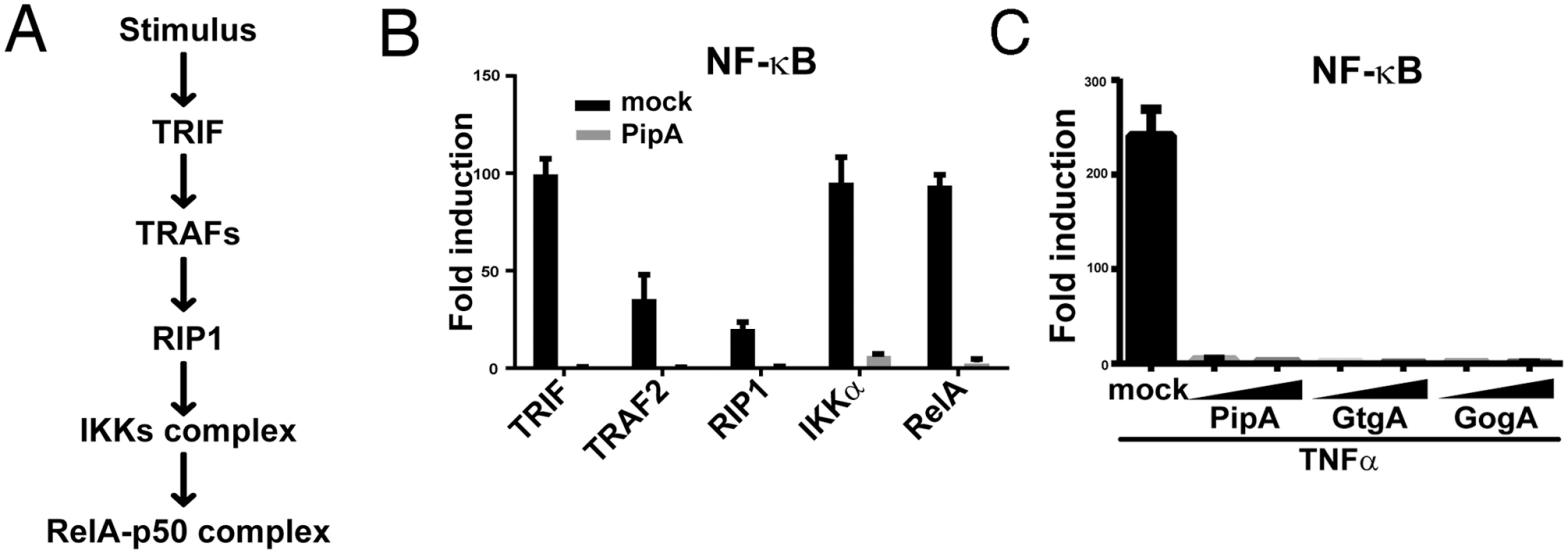
The PipA family of effector proteins can directly inhibit NF-κB signaling. (**A**) Simplified diagram of the TRIF signaling pathway. (**B**) HEK293T cells were transfected with a plasmid encoding a NF-κB reporter construct along with plasmids encoding the indicated proteins (horizontal axis) in conjunction with a plasmid encoding PipA or the empty vector (mock). The reporter activity was subsequently measured 18 hrs after transfection. Data are shown relative to the activity of the reporter in control cells (transfected with the empty vector) and represent the mean ± standard deviation of three independent measurements. (**C**) HEK293T cells were transfected with a plasmid encoding a NF-κB reporter construct along with 25 or 50 ng of a plasmid encoding PipA, GtgA or GogA. Eighteen hours after transfection, cells were treated with TNFα (10 ng/ml) or infected with the Δ*pip*A Δ*gog*A Δ*gtg*A *S*. Typhimurium at a MOI = 5 and the reporter activity was measured 8 hs after treatment. Data are shown relative to the activity of the reporter in uninfected control cells and represent the mean ± standard deviation of three independent measurements.

We then investigated whether the PipA family members by themselves were able to inhibit NF-κB signaling in a context different from bacterial infection [29]. We therefore examined the ability of the PipA family of effectors to prevent NF-κB activation by the transient expression of TRIF, an adaptor for Toll-like receptors [30] (Fig 4A), or by the addition of TNFα, both known potent activators of this pathway. We found that transient expression of the PipA family of effector proteins completely abolished TRIF- (Fig 4B) or TNFα-induced (Fig 4C) NF-κB activation. These results indicate that these effector proteins are not only necessary but also sufficient to inhibit NF-κB signaling and that inhibition occurs even when this pathway is activated by agonists other than *S*. Typhimurium. We also investigated where in the NF-κB signaling pathway these effectors might exert their function by examining the effect of PipA expression on the activation of NF-κB by downstream components of the TRIF signaling pathway [31] (Fig 4A). We found that expression of PipA inhibited an NF-κB-dependent reporter when activated by the expression of TRAF2, RIP1, IKKα, or even the transcription factor RelA itself (Fig 4B). These results indicate that PipA, and by extension the other members of this protein family, must exert their function at the level of or downstream from RelA.

### PipA, GtgA and GogA inhibit the NF-κB pathway by directly targeting RelA

**Fig 5:**
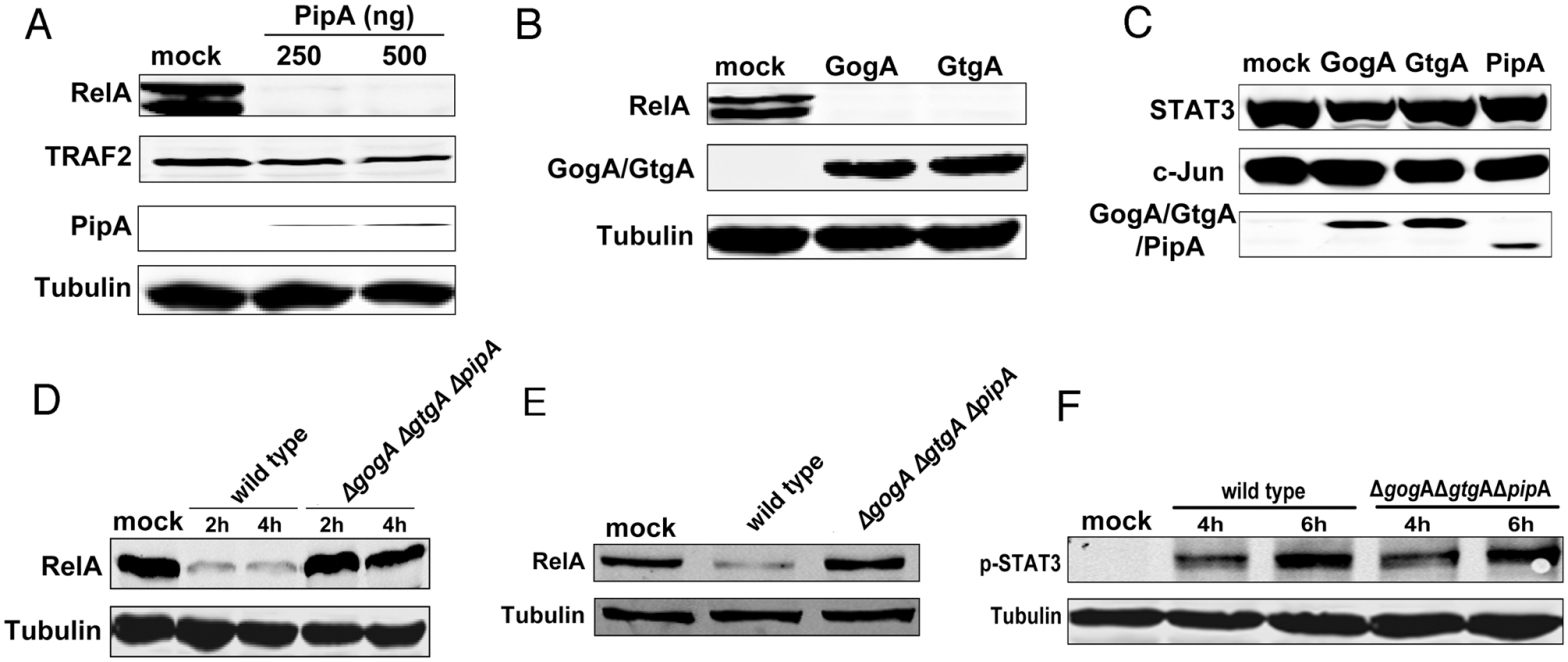
PipA, GtgA and GogA inhibit the NF-κB pathway by targeting RelA. (**A**) HEK293T cells were transiently transfected with plasmids encoding M45-tagged RelA or HA-tagged TRAF2, along with the indicated amounts of a plasmid encoding FLAG-tagged PipA or the empty vector (mock). Eighteen hours after transfection, cell lysates were analyzed by Western blot with anti-M45, anti-HA, anti-FLAG, and anti tubulin antibodies (as loading control). (**B**) HEK293T cells were transiently transfected with plasmids encoding M45-tagged RelA, along with plasmids encoding FLAG-tagged GogA, or GtgA or the empty vector (control). Eighteen hours after transfection, cell lysates were analyzed by Western blot with anti-M45 and anti-FLAG antibodies. (**C**) HEK293T cells were transiently transfected with plasmids encoding M45-tagged STAT3, or c-Jun along with plasmids encoding FLAG-tagged PipA, GogA, or GtgA or the empty vector (mock). Eighteen hours after transfection, cell lysates were analyzed by Western blot with anti-M45 and anti-FLAG antibodies. (**D**) HEK293T cells were transiently transfected with a plasmid encoding M45-tagged RelA. Eighteen hours after transfection, cells were infected with wild type or the *ΔpipA ΔgogA ΔgtgA S*. Typhimurium strains with a MOI = 10. Cell lysates were analyzed by western blot with anti-M45 or anti tubulin (as loading control) antibodies at the indicated times after infection. (**E**) HeLa cells were infected with wild type or the Δ*pip*A Δ*gog*A Δ*gtg*A *S*. Typhimurium strains with a MOI = 10. 6 hs after infection, cell lysates were analyzed by Western blot with anti-RelA, and anti-tubulin antibodies (as loading control). (**F**) HeLa cells were infected with wild type or the Δ*pip*A Δ*gog*A Δ*gtg*A *S*. Typhimurium strains with a MOI = 10. At indicated times after infection, cell lysates were analyzed by Western blot with anti phosphorylated STAT3 and anti-tubulin antibodies (as loading control).

The observation that PipA exerts its function at the level of or downstream from RelA prompted us to investigate the effect of PipA on RelA expression by transiently co-expressing differentially epitope-tagged PipA and RelA. We found that expression of PipA led to a drastic reduction in the levels of RelA expression although it did not alter the expression of TRAF2 (Fig 5A). Similar results were obtained after co-expression of GtgA or GogA (Fig 5B). Expression of PipA, GtgA or GogA did not alter the levels of other transcription factors activated by *Salmonella* infection such as c-Jun or STAT3 (Fig 5C). We then examined the levels of RelA after *S*. Typhimurium infection. We found that, consistent with the transient expression experiments, infection of cultured cells with wild-type *S*. Typhimurium resulted in a marked decreased in the levels of RelA (Fig 5D and 5E). In contrast, infection with the *S*. Typhimurium *ΔgogA ΔgtgA ΔpipA* mutant strain did not alter the levels of RelA in infected cells (Fig 5D and 5E). Furthermore and as previously shown, infection of cultured cells did not alter the endogenous levels of other signaling components including Erk [9–11, 28, 32, 33], cJun [9, 33] [10, 11], p38 [9–11], or STAT3 [28] (Fig 3F), which are robustly activated by *S*. Typhimurium [9–11, 28, 32, 33]. These results indicate that the PipA family of effector proteins inhibits NF-κB signaling by redundantly targeting RelA to block its expression or to stimulate its degradation.

### The PipA-family of effector proteins localize to the nucleus of infected cells

**Fig 6:**
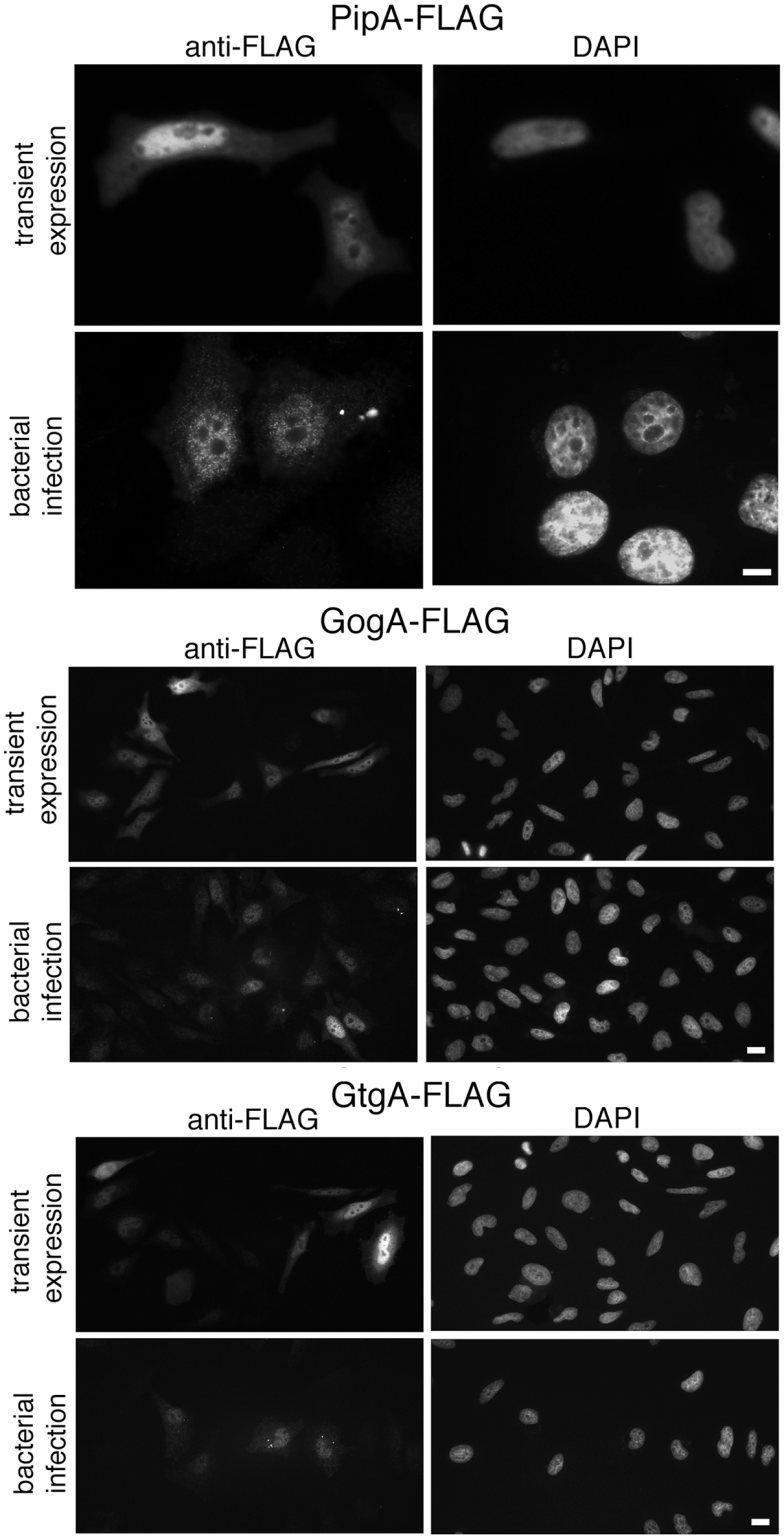
The PipA family of effector proteins localizes to the nucleus of infected cells. Immunofluorescence staining of HeLa cells transfected with a plasmid encoding FLAG-tagged PipA, GogA, or GtgA or infected with *S*. Typhimurium encoding FLAG-tagged PipA, GogA, or GtgA. Eighteen hours after transfection and 4 hours after bacterial infection, cells were stained with an anti FLAG antibody (to visualize the effector proteins) and 4',6-diamidino-2-phenylindole (DAPI) to visualize nuclear DNA. Scale bars: 5 and 10 μm for PipA and GogA/GtgA, respectively.

To gain insight into the mechanism by which the PipA family of effectors targets RelA, we examined the localization of PipA, GtgA, and GogA both, after transient transfection or bacterial infection. We found that these effectors localized to the nucleus both after transient expression or bacterial infection (Fig 6). These results indicate that these effectors most likely target RelA subsequent to activation of the NF-κB signaling pathway, which results in the nuclear translocation of the transcription factors. This observation is consistent with the kinetics of the PipA/GtgA/GogA-dependent inhibition of NF-κB during bacterial infection (Fig 3D) since the phenotype was only apparent later in infection and subsequent to the *Salmonella*-induced NF-κB activation.

### PipA, GtgA and GogA are specific proteases for transcription factors of the RelA family

**Fig 7:**
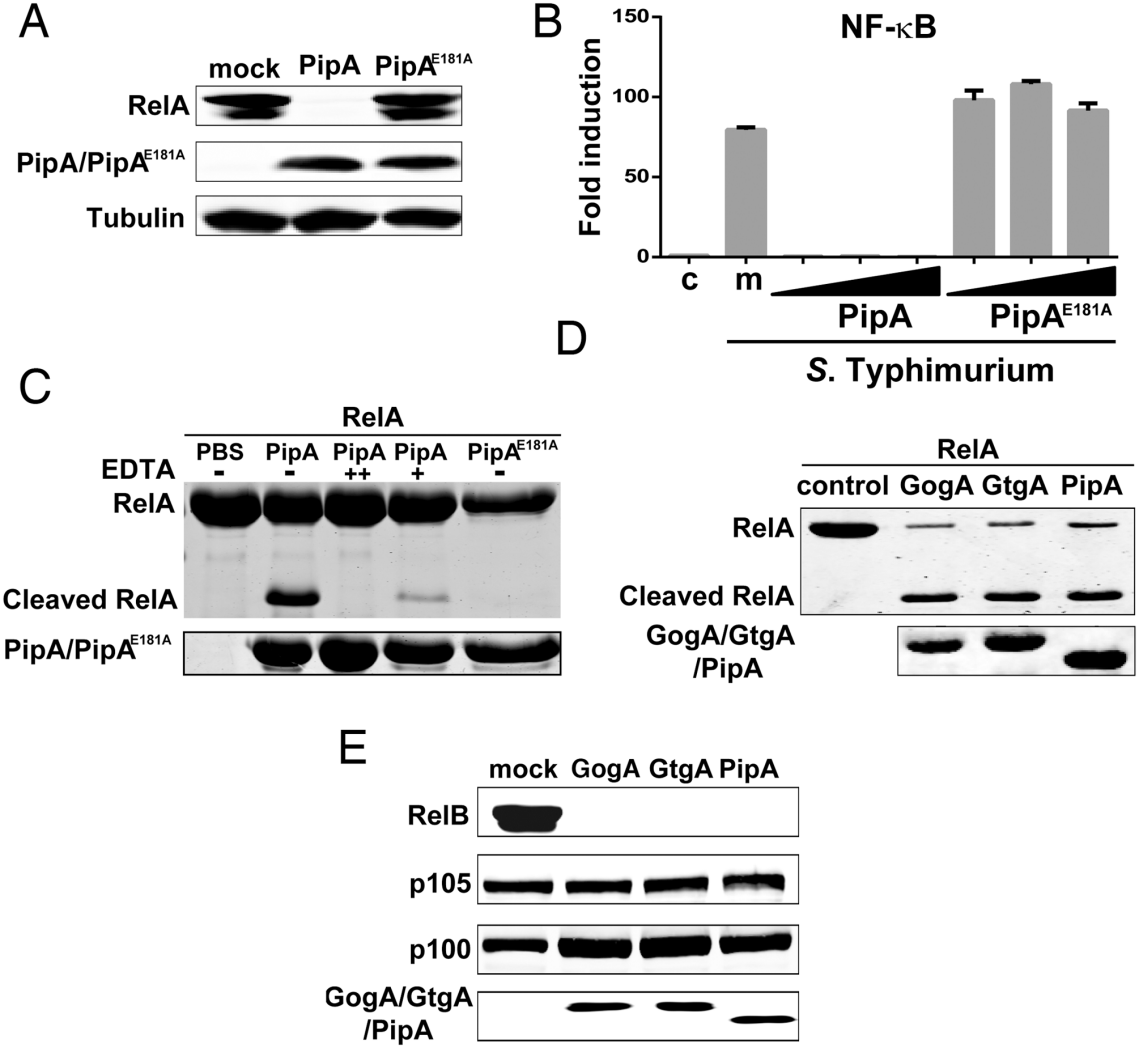
PipA, GtgA and GogA are specific proteases for transcription factors of the RelA family. (**A**) HEK293T cells were transiently transfected with plasmids encoding M45-tagged RelA, along with plasmids encoding either FLAG-tagged PipA or its catalytic mutant PipA^E181A^. Eighteen hours after transfection, cell lysates were analyzed by Western blot with anti-M45, anti-FLAG, and anti-tubulin (as loading control) antibodies. (**B**) HEK293T cells were transiently transfected with a plasmid encoding a NF-κB reporter construct, along with 25, 50 and 100 ng of plasmid DNA encoding either FLAG-tagged PipA or its catalytic mutant PipA^E181A^. Eighteen hours after transfection, cells were infected with the Δ*gog*A/Δ*gtg*A/Δ*pip*A *S*. Typhimurium mutant strain at MOI = 5 and the reporter activity measured 8 hs after infection. Data are the reporter activity relative to control cells (no infection) and represent the mean ± standard deviation of three independent experiments. (**C**) Purified RelA_1-210_ (8μg) was incubated with purified PipA or PipA^E181A^ (2.5 μg) in a reaction buffer (40 μl) in the presence [50 (+) or 100 (++) mM] or absence (-) of EDTA. The reaction was stopped by addition of SDS loading buffer, proteins separated by SDS—PAGE and visualized by Coomassie blue staining. RelA_1-210_ and its cleaved product are indicated. (**D**) Purified RelA_1-210_ was incubated with purified GogA, GtgA, or PipA in a reaction buffer. The reaction was stopped by addition of SDS loading buffer, proteins separated by SDS—PAGE and visualized by Coomassie blue staining. RelA_1-210_ and its cleaved product are indicated (note that differences in cleavage efficiency between panels C and D are likely the result of differences in the solubility of the RelA preparation, which tended to aggregate after even limited storage). (**E**) HEK293T cells were transiently transfected with plasmids encoding M45-tagged RelB, p105, or p100, along with plasmids encoding FLAG-tagged PipA, GogA, or GtgA or the empty vector (mock). Eighteen hours after transfection, cell lysates were analyzed by western blot with anti-M45 and anti-FLAG antibodies.

Primary amino acid sequence similarity searches did not yield proteins with significant similarity to the PipA family of effectors other than true homologs in other bacteria. However, structural similarity searches identified features present in metalloproteinases such as a region that fits the consensus for the zinc-binding site that is a signature for these proteinases and that is essential for their catalytic activity (S8 Fig). We therefore introduced mutations in the predicted zinc-binding site of PipA and examined the effect of this mutation on the expression of RelA. Introduction of this mutation effectively prevented the ability of PipA to reduce the levels of RelA (Fig 7A) or inhibit *Salmonella*-induced NF-κB signaling (Fig 7B) in transient transfection experiments.

To ascertain if the observed proteolytic degradation of RelA was the direct result of the proteolytic activity of the PipA family of effector proteins, we purified PipA and the PipA^E181A^ catalytic mutants and examined their proteolytic activity towards purified RelA *in vitro*. We found that PipA, but not the PipA^E181A^ catalytic mutant, was able to cleave RelA and cleavage occurred in the presence but not in the absence of divalent cations (Fig 7C and 7D). To identify the precise cleavage site we determined the amino-terminal sequence of the RelA cleavage product after PipA digestion. We found that PipA cleaves between residues Gly40 and Arg41 of RelA (S1 Table). The cleavage products, which are presumably degraded *in vivo*, are nonetheless expected to be non functional since they would lack critical domains necessary for transcriptional activity [34]. These results indicate that the PipA family of effector proteins are proteases that directly target RelA.

In addition to RelA (p65), there are other members of the NF-κB family of transcription factors that share the Rel-homology domain and form homo or heterodimers with one another thus adding complexity to the transcriptional outputs [35]. We therefore tested whether the PipA family of effectors could target other members of the NF-κB family. We found that PipA, GtgA and GogA were able to effectively cleave RelB but did not cleave p100 (NF-κB2) or p105 (NF-κB1) under the experimental conditions used in these studies (Fig 7E). These results indicate that the PipA family of effectors are proteases that selectively target a subset of the NF-κB transcription factors.

## Discussion

It is often overlooked that host-pathogen interactions shaped by long-standing associations have evolved not just to maximize pathogen replication but also to preserve host homeostasis. *S*. Typhimurium, for example, can induce its own internalization into non-phagocytic cells through the activity of the effector proteins SopE and SopE2, which are exchange factors and thus activators of Rho-family GTPases [14, 36]. These responses are subsequently reversed by the delivery of SptP, an effector with an opposing GAP activity [15]. Similarly, other effectors oppose *Salmonella*-induced signaling pathways leading to nuclear responses [17, 37]. We report here the discovery of a family of effector proteins that proteolytically targets a subset of NF-κB transcription factors thus inhibiting a key signaling pathway in the inflammatory responses to microbial pathogens. The evolution of specific proteases to target key cellular processes is an emerging theme in bacterial pathogenesis [20, 38, 39]. In fact, some strains of *E*. *coli* have been shown to target the NF-κB signaling pathway utilizing NleC, a Zinc-metalloprotease that is also delivered by their type III secretion systems but that is not a homolog of the proteins described in this study [40–43]. NleC has been reported to cleave p65 between Pro10 and Ala11 or between Cys38 and Glu39, which is in contrast to the PipA family of Salmonella effectors, which cleave RelA between residues Gly40 and Arg41. In any case, all cleavage sites for these effectors are located within a flexible loop of RelA [44].

*S*. Typhimurium is known to potently activate the NF-κB signaling pathway to induce intestinal inflammation [9–11], which is required for its ability to acquire essential nutrients in the gut [12, 13]. We have shown here that absence of the PipA, GtgA, and GogA effectors resulted in increased lethality that, we hypothesize, may be due to heightened production of pro-inflammatory cytokines in the gut. Consistent with this hypothesis we did not observe increased lethality when the mutant was administered systemically and we only observed an increase in the levels of some pro-inflammatory cytokines in the sera and pro-inflammatory cytokine mRNAs in intestinal tissues in animals that have been orally infected with the mutant strain. We therefore conclude that *S*. Typhimurium has evolved these effector proteins to preserve host homeostasis even if at the expense of decreasing its potential virulence.

It is well established that in the mouse, the divalent metal ion transporter NRAMP1 (SLC11A1) confers resistant to *S*. Typhimurium infections [27]. Thus, NRAMP1-deficient animals are ~1,000 fold more susceptible to *S*. Typhimurium regardless the inoculation route. Therefore it is noteworthy that the hypervirulent phenotype of the *S*. Typhimurium *ΔgogA ΔgtgA ΔpipA* mutant strain is paradoxically manifested only in wild type animals, which are more resistant to infection. However, our results indicate that the increased NF-κB activation stimulated by the infection with the mutant strain does not result in higher bacterial loads but rather in a significant increase in the levels of pro-inflammatory cytokine in the intestine. We hypothesize that the early death observed in a significant proportion of the animals infected with the *S*. Typhimurium *ΔgogA ΔgtgA ΔpipA* mutant strain may be the result of a “cytokine storm” triggered by the increased cytokine production in the intestine of the wild type animals infected by the mutant. Consistent with this hypothesis we only observed the increased virulence phenotype after oral administration of the bacterial mutant strains but not after intraperitoneal infection. Previous studies have indeed shown that the intestinal inflammatory response to *S*. Typhimurium early during infection is higher in *nramp1* ^+/+^ than in *nramp1* ^-/-^ mice [45], which is consistent with our hypothesis. Why the presence of NRAMP1 (SLC11A1) results in an increased inflammatory response to *Salmonella* is unclear but it is intriguing that previous studies have shown that this transporter modulates signal transduction pathways leading to inflammation [46, 47].

It is widely believed that the remarkable advances in the understanding of the mechanisms of pathogenesis of the most important pathogens will result in the development of novel antimicrobial strategies aimed at specifically targeting those mechanisms. In this context, type III secretion system machines and their effector proteins are viewed as potential targets for the development of such type of drugs [48]. However, the findings reported here showing that absence of a family of effector proteins results in increased virulence indicates that caution should be exercised before targeting biochemical activities of effectors whose functions have not been thoroughly characterized. In summary, these finding revealed a remarkable adaptation of a bacterial pathogen to secure its own long-term survival.

## Materials and Methods

### Plasmids

All plasmids used in these studies are listed in S2 Table. Human TRIF, TRAF2, RIP1, IKKα, p105 or RelA were amplified from a human cDNA library and cloned into the vectors pCMV-3XHA, pRK5-M45 or pET15b to generate HA-tagged versions of TRIF, TRAF2, RIP1, IKKα, and RelA, M45-tagged versions of p105 and RelA, and His-tagged RelA (all tags were located at the amino terminus of the respective proteins). *gogA*, *gtgA* and *pipA* were amplified from the chromosome of *S*. Typhimurium strain SL1344 and cloned into the vectors pRK5-Flag or pET15b to generate Flag-tagged or His-tagged versions of the proteins. For *S*. Typhimurium mutant complementation studies, *gogA*, *gtgA* or *pipA* were cloned into the pBAD24 vector placing their expression under the control of an arabinose inducible promoter [49]. The plasmids pCMV4-human p100 and pCDNA3-mouse RelB were purchased from Addgene and were subcloned into the vector pRK5-M45 to generate M45-taggged versions of these proteins. The catalytic mutant PipA^E181A^ was generated using PCR-mediated mutagenesis. Plasmids encoding Gal4-Elk1 and Gal4-luc were provided by Dr. Feng Shao (National Institute of Biological Sciences, Beijing). Plasmids encoding M45-tagged STAT3 and c-Jun were provided by Dr. Walther Mothes (Yale School of Medicine, Yale University).

### *S*. Typhimurium strains and growth condition

All of *S*. Typhimurium strains were derived from *S*. Typhimurium strain SL1344 [50] and are listed in S2 Table. Bacterial mutants were constructed by allelic exchange as previously described [51]. *S*. Typhimurium strains were cultured in LB containing 0.3M NaCl to induce the expression of the SPI-1 T3SS [52].

### Cell culture and bacterial infection

The human embryonic kidney epithelial HEK 293T (ATCC) and human HeLa (ATCC) and epithelial Henle-407 (Roy Curtiss laboratory collection) cell lines were cultured in antibiotic free Dulbecco’s Modified Eagle Medium (DMEM, Gibco) supplemented with 10% bovine calf (Henle-407) or bovine fetal (HEK293T) sera. For bacterial infections, 2 x 10^5^ HEK293T cells or 7 x 10^4^ Henle-407 or HeLa cells were seeded into each well of a 24-well plate. Eighteen hours later, cells were infected for 1 hr with the different *S*. Typhimurium strains at the multiplicities of infection (MOI) indicated in the figure legends. Cells were then treated with gentamicin (100 μg/ml) for 1 hour to kill extra cellular bacteria. In experiments involving longer infection times, the infected cells were cultured in medium with low concentration gentamicin (10 μg/ml) for the indicated times.

### Immunofluorescence staining

HeLa cells (ATCC) grown on glass coverslips were transiently transfected using Lipofectamine 2000 (Invitrogen) with 0.5 μg of plasmid encoding FLAG-epitope-tagged PipA or infected with *S*. Typhimurium strain expressing FLAG-tagged PipA, GogA, or GtgA as described above. Eighteen hours after transfection and 4 hours after bacterial infection, cells were washed once with PBS and fixed in 4% PFA/PBS for 20 min at RT. FLAG-tagged PipA, GtgA, or GogA were stained with mouse monoclonal anti-FLAG M2 (Sigma; 1:10,000 dilution) and secondary anti-mouse antibody conjugated to Alexa 488 (Invitrogen, 1:1,000). Images were acquired with an inverted microscope (Eclipse TE2000-U; Nikon) equipped with a CCD camera (MicroMAX RTE/CCD-1300Y; Princeton Instruments).

### Bacteria internalization and intracellular replication

Cultured epithelial Henle-407 cells grown in a 24-well plate were infected for 1 h and incubated in the presence of gentamicin as described above. Thirty minutes or 8 hs after gentamicin treatment, cells were washed twice with HBSS and then lysed in 300 μl 0.1% Sodium Deoxycholate (DOC) in HBSS to release their bacterial content. Multiple dilutions were then plated onto LB plates containing streptomycin to determine colony forming units (c.f.u.).

### Luciferase reporter assay

Dual luciferase assay was performed following the manufacturer’s instructions (Promega). To monitor the activation of the Erk pathway, HEK293T cells plated in 24-well plates were co-transfected with 0.4 μg of Gal4-Elk1, 0.4 μg of Gal4-luc, 20 ng of pRL-Actin as internal control. To measure NFκB or STAT3 activation, HEK293T cells were co-transfected with 20 ng of the pGl3-luc reporter plasmid encoding a NF-κB- or a STAT3-responsive element and 20 ng of pRL-actin as internal control. Eighteen hs after transfection, cells were infected with bacteria or were lysed to measure luciferase activity as previously described[53].

### *In vitro* protease assay

Purified His-tagged-RelA_1-210_ was mixed with purified His-tagged-GogA, His-tagged-GtgA, His-tagged-PipA or His-tagged-PipA^E181A^ in 40 μl of reaction buffer (50mM Tris-HCl pH 7.5, 2mM CaCl_2_, 50mM NaCl) in the absence of EDTA or in the presence of EDTA. Reactions were carried out for 1 hr at room temperature and stopped by the addition of SDS loading buffer. Digestion products were analyzed by SDS-PAGE followed by Coomassie Blue staining. When indicated, bands were transferred to PDVF membranes and subjected to amino terminal amino acid sequencing (performed at Molecular Structure Facility, University of California, Davis).

### Quantitative PCR

Total RNA from mouse tissues (cecum) were isolated using TRIzol (Invitrogen) reagent according to the manufacture’s protocol and were reversed-transcribed with the iScript reverse transcriptase (BIORAD). Quantitative PCR was performed using iQ SYBR Green Supermix (BIORAD) in an iCycler real time PCR machine (Bio-Rad) with the following primers:

GAPDH fw ATTGTCAGCAATGCATCCTG

GAPDH re ATGGACTGTGGTCATGAGCC

TNFα fw CCACCACGCTCTTCTGTCTAC

TNFα re AGGGTCTGGGCCATAGAACT

KC re TCTCCGTTACTTGGGGACAC

KC fw ACCCAAACCGAAGTCATAGC

IL1β fw GCAACTGTTCCTGAACTCAACT

IL1β re ATCTTTTGGGGTCCGTCAACT

MIP1α fw ACCATGACACTCTGCAACCA

MIP1α re GTGGAATCTTCCGGCTGTAG

### Mouse infections

All animal experiments were conducted according to protocols approved by Yale University’s Institutional Animal Care and Use Committee. A C57BL/6 mouse line carrying a wild type allele of *Nramp1* (*Slc11a1*) (from the 129/Svj mouse) was generated by backcrossing to C57BL/6 for 12 generations. The *Nramp1* (*Slc11a1*) alleles were verified by PCR amplification and nucleotide sequencing as previously described [54]. Groups of age- and sex-matched C57BL/6 *Nramp1* -/- and C57BL/6 *Nramp1* +/+ mice were infected at 8–12 week of age. For oral infections food was removed 4 hs prior to inoculation, and mice were administered (by stomach gavage) 100 μl of 10% bicarbonate solution (to buffer the stomach pH) followed by the indicated bacterial dose in 100 μl PBS. For intraperitoneal injection, the indicated bacterial dose was administered in 100 μl PBS. To determine bacterial loads, tissues were mechanically homogenized in 3 ml PBS containing 0.05% sodium deoxycholate, and dilutions were plated on LB plates containing streptomycin to determine colony-forming units as previously described [54].

### Histology

Histopathological analysis of intestinal tissues was carried out as previously described [54]. Briefly, a portion of the distal cecal tip of experimental animals was fixed in 3.7% formalin for 72 h, then transferred to 70% ethanol for 48 h prior to paraffin embedding, sectioning, and hematoxylin-eosin staining. Pathology scores were determined as previously described [55].

### Ethics statement

All animal experiments were conducted according to protocols approved by Yale University’s Institutional Animal Care and Use Committee under protocol number 2013–07858. The IACUC is governed by applicable Federal and State regulations, including those of the Animal Welfare Act (AWA), Public Health Service (PHS), and the United States Department of Agriculture (USDA) and is guided by the U.S. Government Principles for the Utilization and Care of Vertebrate Animals Used in Testing, Research and Training.

## Supporting Information

S1 Fig Amino acid sequence alignment of the *S*. Typhimurium PipA family of effector proteins. GtgA, GogA, and PipA are from *S*. Typhimurium strains SL1344. The Escherichia coli (WP_044710484.1) and Arsenophonus nasoniae (WP_026822997.1) sequences were obtained from National Center for Biotechnology Information data base.
(TIF)

S2 Fig Absence of the PipA-family of effector proteins does not increase the bacterial loads of *S*. Typhimurium in NRAMP1-deficient mice. C57BL/6 (*nramp1* ^*-/-*^) mice were orally (**A**) or intraperitoneally (**B**) infected with wild-type *S*. Typhimurium or the *ΔpipA/ΔgogA/ΔgtgA* mutant and bacterial loads in the indicated tissues enumerated 6 days after infection. Each circle represents the bacterial load for an individual animal and horizontal bars indicate geometric means.
(TIF)

S3 Fig Absence of the PipA-family of effector proteins does not increase the bacterial loads of *S*. Typhimurium. C57BL/6 (*nramp* ^*+/+*^) mice were orally infected with wild-type *S*. Typhimurium or the *ΔpipA/ΔgogA/ΔgtgA* mutant and bacterial loads in the indicated tissues enumerated 15 days after infection. Each circle represents the bacterial load for an individual animal and horizontal bars indicate geometric means. The differences between the means of the wild type and mutant cfu in the different tissues were not statistically significant (*p* > 0.5).
(TIF)

S4 Fig Survival of *nramp1* ^*+/+*^ or *nramp1* ^*-/-*^ mice after oral infection with the *S*. Typhimurium *ΔpipA ΔgogA ΔgtgA* mutant strain. C57BL/6 *nramp1* ^+/+^ (**A**) or C57BL/6 *nramp1* ^-/-^ (**B**) mice were orally infected with 5 x 10^8^ c. f. u. of wild type *S*. Typhimurium (n = 10) or the *ΔpipA/ΔgogA/ΔgtgA* triple mutant (n = 11) and survival was recorded over time. The *p* values of the difference in the survival of animals infected with wild type or mutant strains are shown.
(TIF)

S5 Fig Serum cytokine levels in mice infected with wild type S. Typhimurium or the Δ*pip*A Δ*gog*A Δ*gtg*A isogenic mutant strain. C57/BL6 *nramp*+/+ mice were orally infected with wild type (n = 4) or Δ*pip*A Δ*gog*A Δ*gtg*A (n = 4) S. Typhimurium strains and 4 days after infection the levels of the indicated cytokines in the serum were measured by ELISA. Values represent the mean ± standard deviations of the measurements.
(TIF)

S6 Fig Absence of GtgA, GogA and PipA results in increased intestinal inflammation. C57BL/6 *nramp1* ^*+/+*^ mice were either mock-infected (control) or infected orally with 10^8^ wild type or *ΔgtgA/ΔgogA/ΔpipA S*. *typhimurium* strains. Four days after infection, ceca were removed, fixed, and embedded in paraffin, and tissue sections were stained with hematoxylin and eosin. Each photomicrograph was obtained from a different animal. Similar results were obtained in four independent animals for each group.
(TIF)

S7 Fig Absence of the PipA-family of type III secretion effector proteins does not affect the ability of *S*. Typhimurium to invade and replicate within cultured epithelial cells. **A**, Henle-407 cells were infected with *S*. Typhimurium wild type, or the Δ*gog*A, Δ*gtg*A, Δ*pip*A, or Δ*gog*A/Δ*gtg*A/Δ*pip*A triple mutant strains at MOI = 5. Bacterial invasion was measured by the gentamicin protection assay as indicated in Material and Methods. Values represent the % of the inoculum that survive the gentamicin treatment due to bacterial internalization and have been standardized relative to the levels of invasion of wild-type *S*. Typhimurium, which was considered to be 100%. The results represent the mean ± standard deviation of three independent experiments. Differences between the values of wild type and the different mutants were not statistically significant (p > 0.05). **B**, Henle-407 cells were infected with *S*. Typhimurium wild type, or the Δ*gog*A, Δ*gtg*A, Δ*pip*A, or Δ*gog*A/Δ*gtg*A/Δ*pip*A triple mutant strains at MOI = 5. The number of CFU was enumerated 30 minutes and 8 hs after infection. Values are the fold change after 8 hs of infection (relative to the values at 30 minutes after infection) and represent the mean ± standard deviation of three independent experiments. Differences between the values of wild type and the different mutants were not statistically significant (p > 0.05).
(TIF)

S8 Fig Conservation of a protease motif in the PipA family of effector proteins. Amino acid sequence alignment of the *S*. Typhimurium PipA family of effector proteins depicting the location of a conserved metalloprotease Zn-binding motif.
(TIF)

S1 Table RelA amino terminal sequence after digestion with PipA, GogA or GtgA, (PDF)

S2 Table Strains and plasmids used in this study, (PDF)
