# Supplementary figures and images for: A Family of Salmonella Type III Secretion Effector Proteins Selectively Targets the NF-κB Signaling Pathway to Preserve Host Homeostasis
Source: PLoS Pathog. 2016 Mar 2;12(3):e1005484. doi: 10.1371/journal.ppat.1005484 (PMC4775039; doi:10.1371/journal.ppat.1005484)

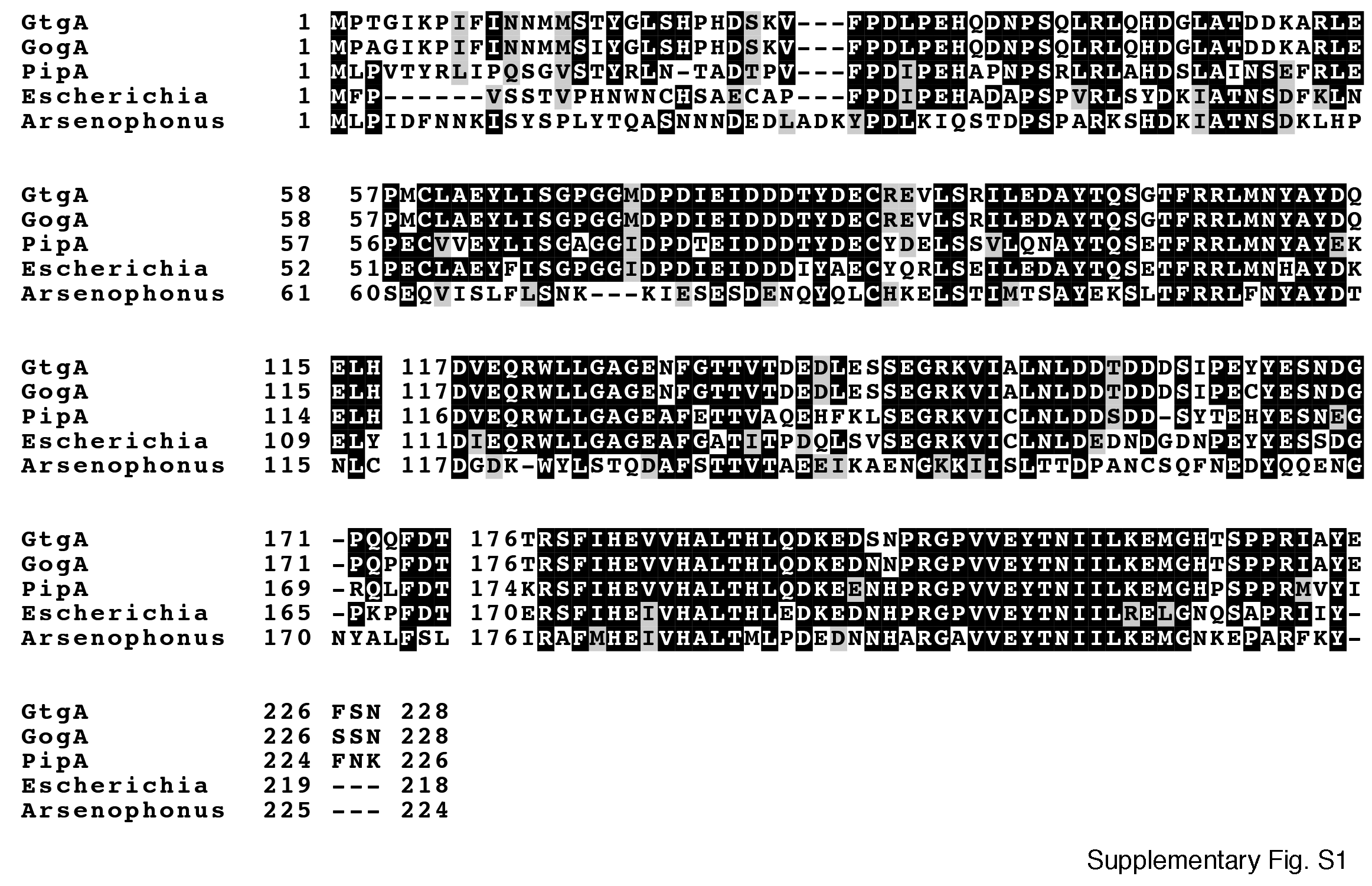

Supplement: S1 Fig — GtgA, GogA, and PipA are from S. Typhimurium strains SL1344. The Escherichia coli (WP_044710484.1) and Arsenophonus nasoniae (WP_026822997.1) sequences were obtained from National Center for Biotechnology Information data base. (TIF) [file ppat.1005484.s001.tif]

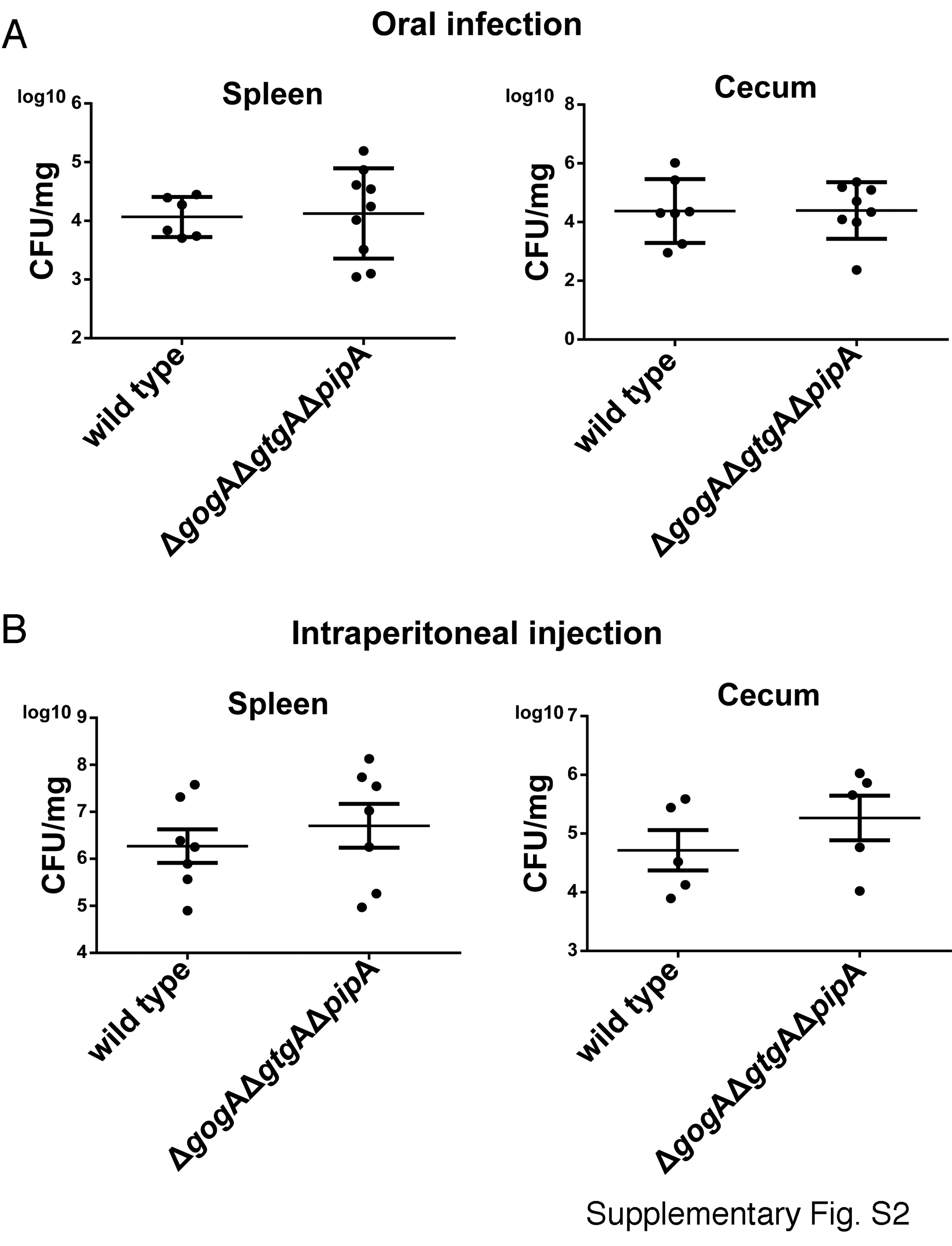

Supplement: S2 Fig — C57BL/6 (nramp1 -/-) mice were orally (A) or intraperitoneally (B) infected with wild-type S. Typhimurium or the ΔpipA/ΔgogA/ΔgtgA mutant and bacterial loads in the indicated tissues enumerated 6 days after infection. Each circle represents the bacterial load for an individual animal and horizontal bars indicate geometric means. (TIF) [file ppat.1005484.s002.tif]

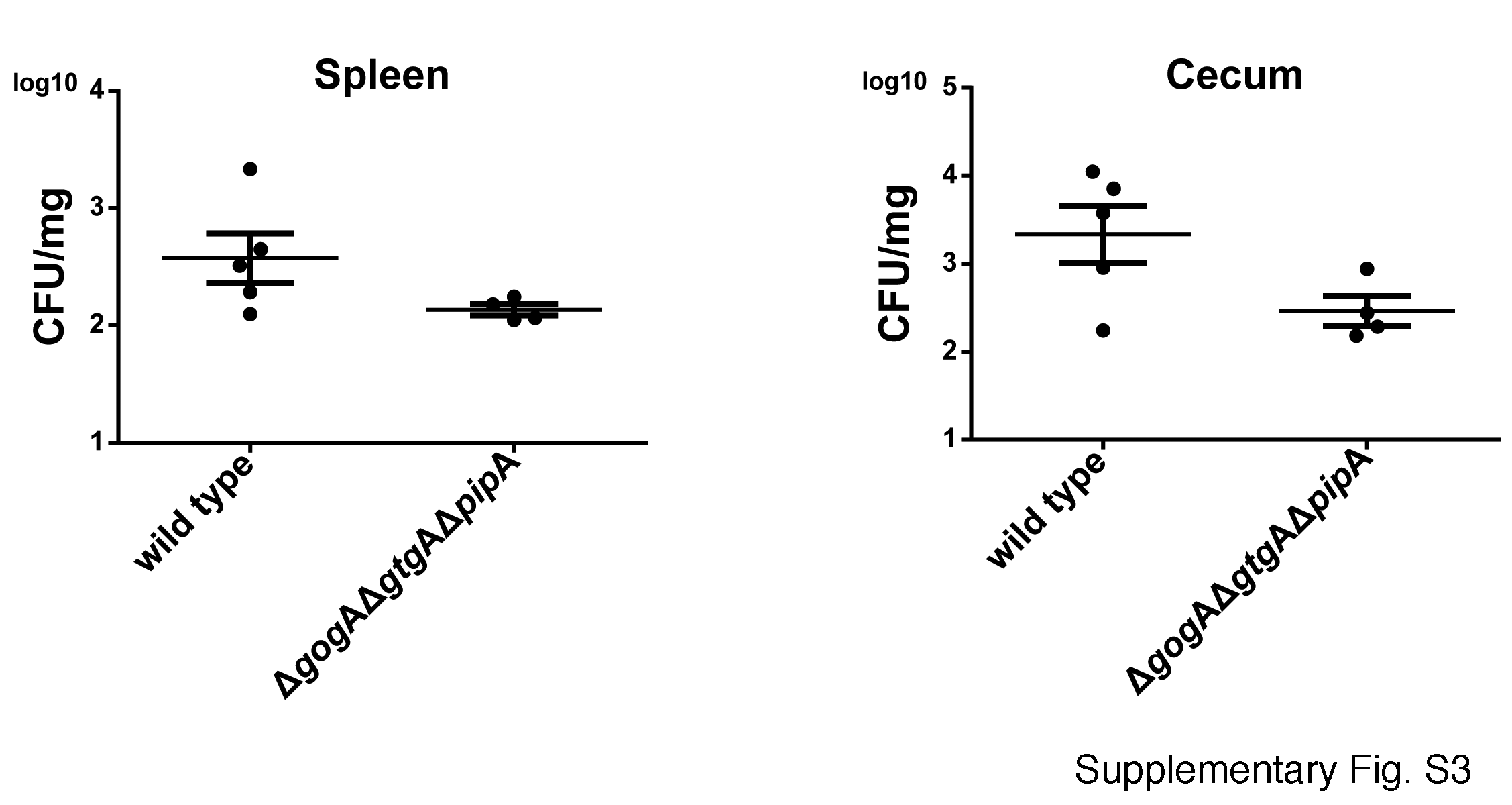

Supplement: S3 Fig — C57BL/6 (nramp +/+) mice were orally infected with wild-type S. Typhimurium or the ΔpipA/ΔgogA/ΔgtgA mutant and bacterial loads in the indicated tissues enumerated 15 days after infection. Each circle represents the bacterial load for an individual animal and horizontal bars indicate geometric means. The differences between the means of the wild type and mutant cfu in the different tissues were not statistically significant (p > 0.5). (TIF) [file ppat.1005484.s003.tif]

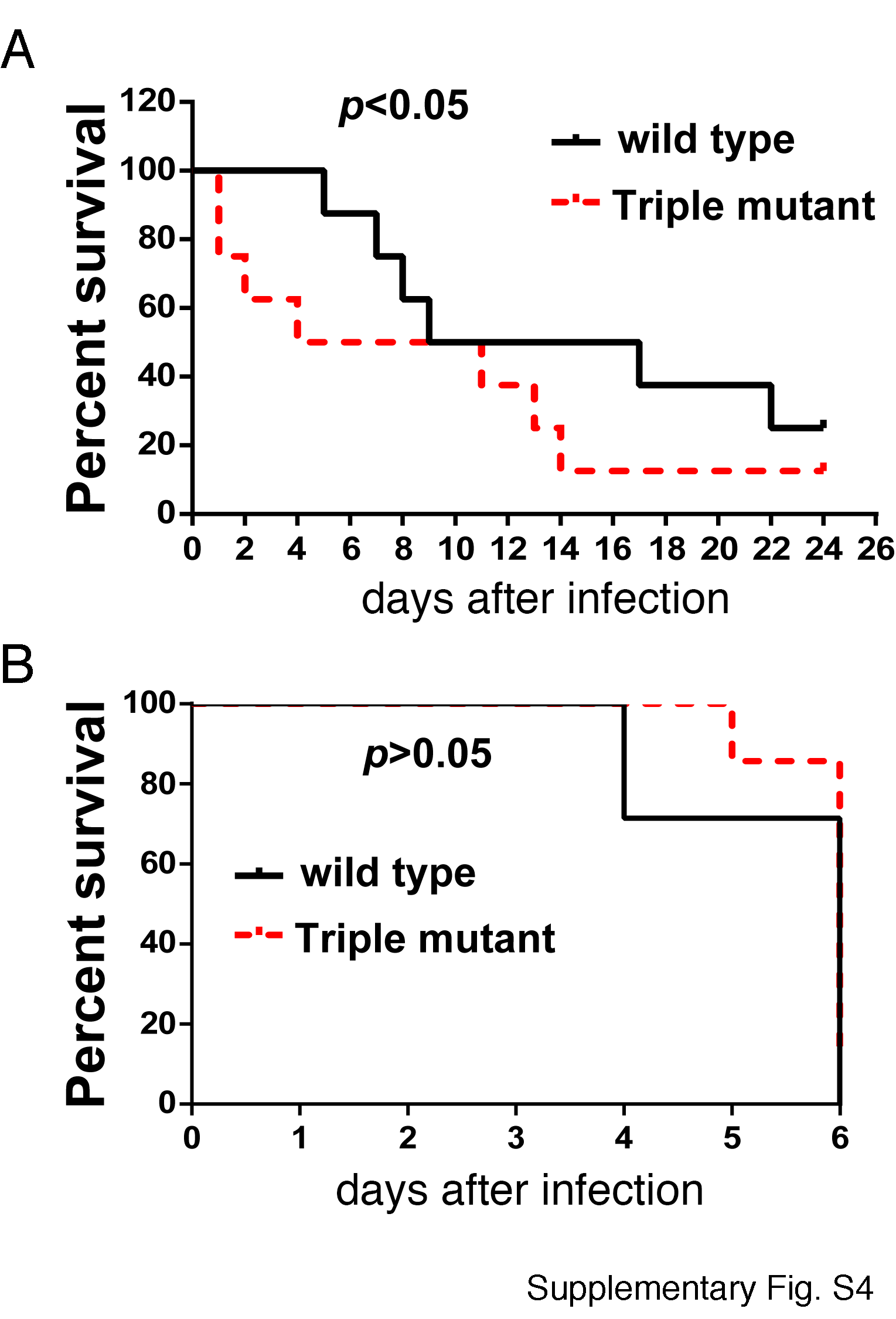

Supplement: S4 Fig — C57BL/6 nramp1 +/+ (A) or C57BL/6 nramp1 -/- (B) mice were orally infected with 5 x 10^8^ c. f. u. of wild type S. Typhimurium (n = 10) or the ΔpipA/ΔgogA/ΔgtgA triple mutant (n = 11) and survival was recorded over time. The p values of the difference in the survival of animals infected with wild type or mutant strains are shown. (TIF) [file ppat.1005484.s004.tif]

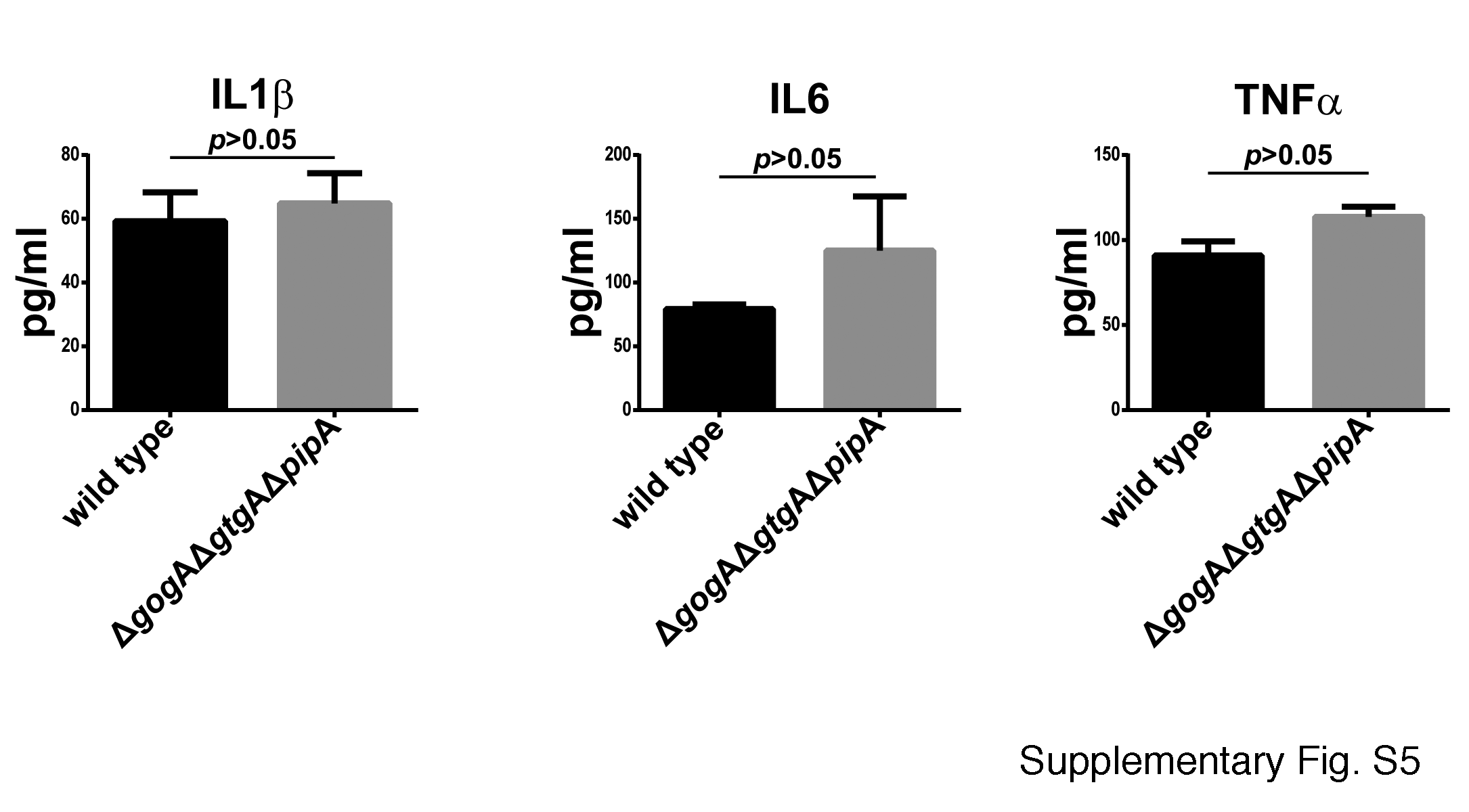

Supplement: S5 Fig — C57/BL6 nramp+/+ mice were orally infected with wild type (n = 4) or ΔpipA ΔgogA ΔgtgA (n = 4) S. Typhimurium strains and 4 days after infection the levels of the indicated cytokines in the serum were measured by ELISA. Values represent the mean ± standard deviations of the measurements. (TIF) [file ppat.1005484.s005.tif]

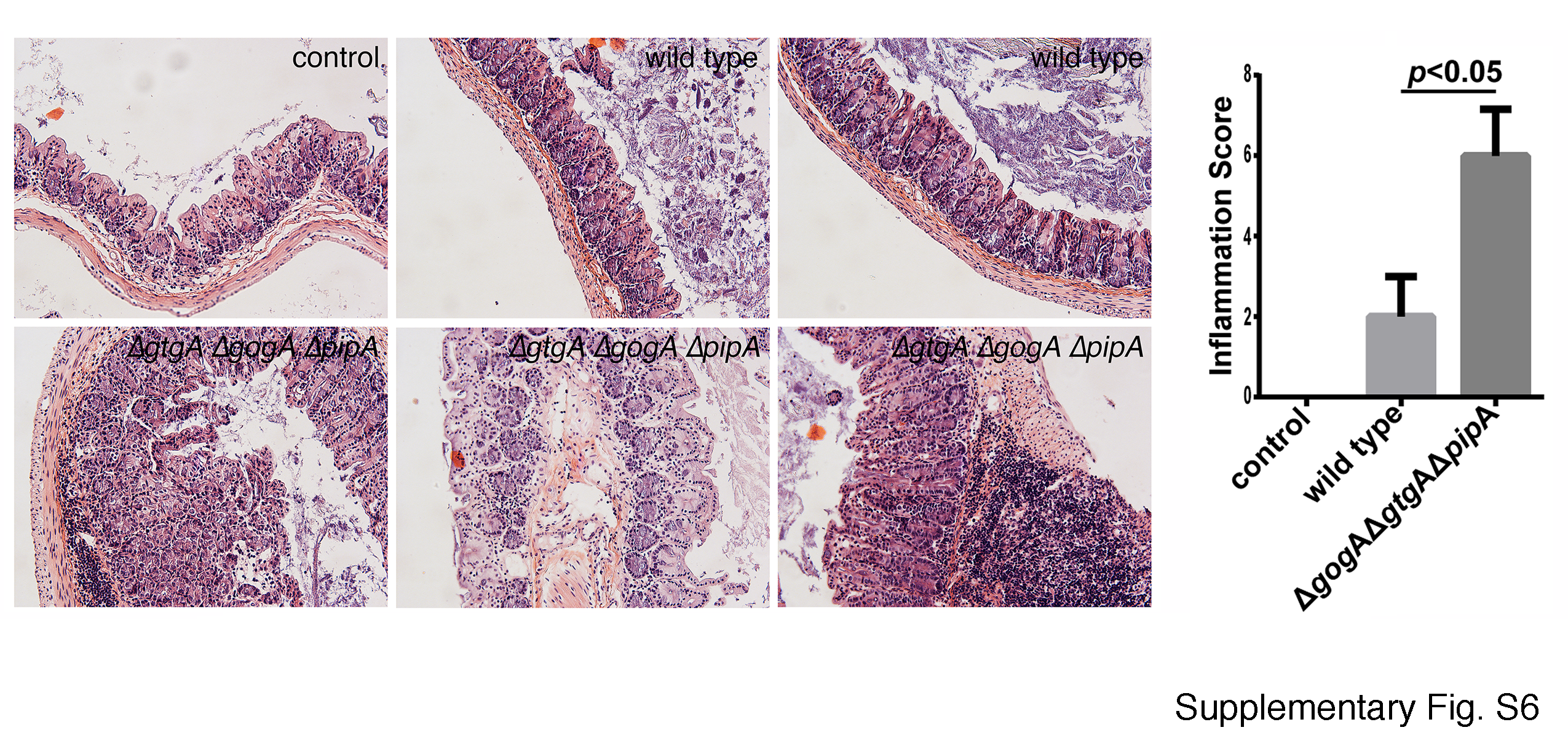

Supplement: S6 Fig — C57BL/6 nramp1 +/+ mice were either mock-infected (control) or infected orally with 108 wild type or ΔgtgA/ΔgogA/ΔpipA S. typhimurium strains. Four days after infection, ceca were removed, fixed, and embedded in paraffin, and tissue sections were stained with hematoxylin and eosin. Each photomicrograph was obtained from a different animal. Similar results were obtained in four independent animals for each group. (TIF) [file ppat.1005484.s006.tif]

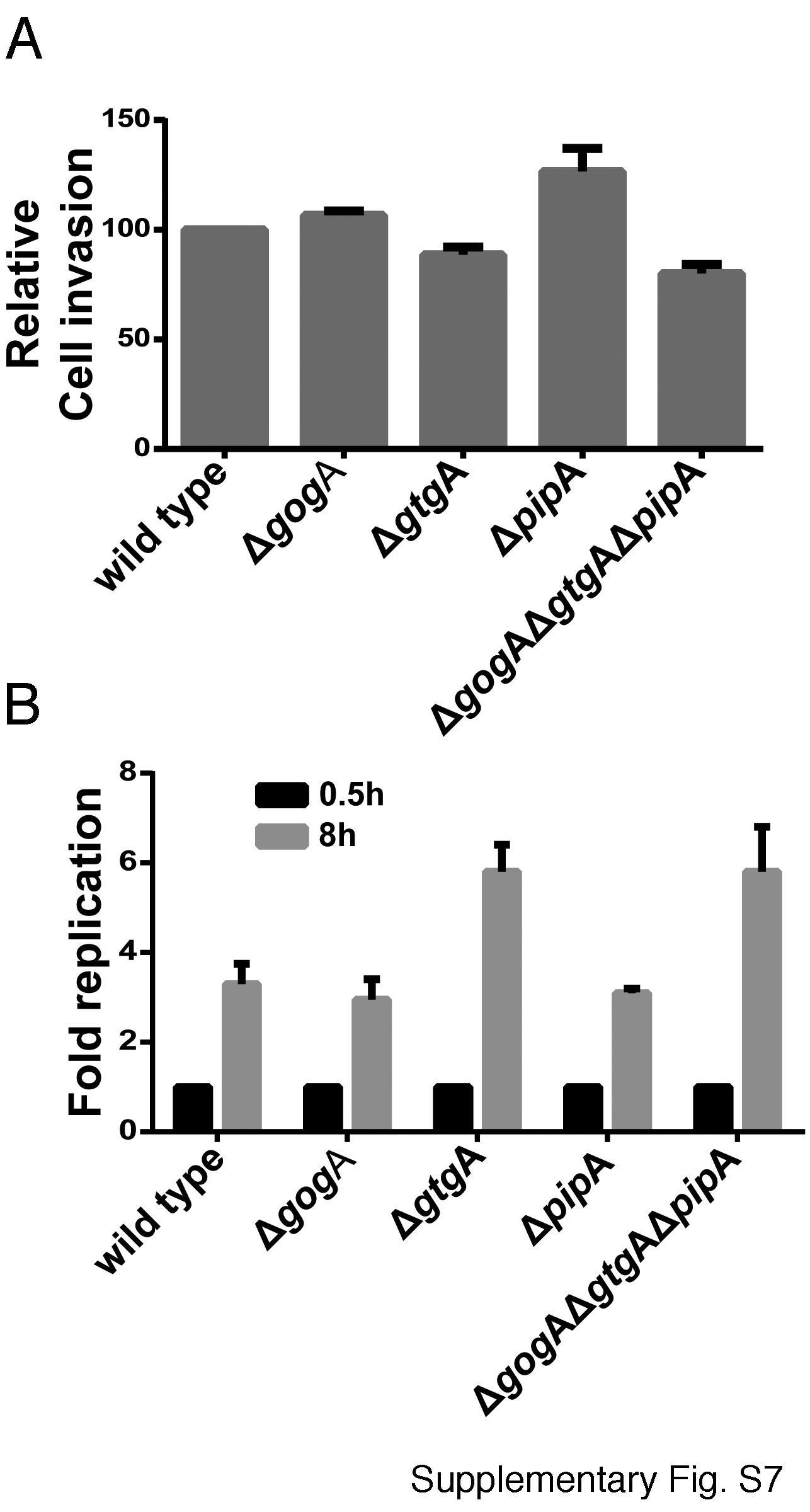

Supplement: S7 Fig — A, Henle-407 cells were infected with S. Typhimurium wild type, or the ΔgogA, ΔgtgA, ΔpipA, or ΔgogA/ΔgtgA/ΔpipA triple mutant strains at MOI = 5. Bacterial invasion was measured by the gentamicin protection assay as indicated in Material and Methods. Values represent the % of the inoculum that survive the gentamicin treatment due to bacterial internalization and have been standardized relative to the levels of invasion of wild-type S. Typhimurium, which was considered to be 100%. The results represent the mean ± standard deviation of three independent experiments. Differences between the values of wild type and the different mutants were not statistically significant (p > 0.05). B, Henle-407 cells were infected with S. Typhimurium wild type, or the ΔgogA, ΔgtgA, ΔpipA, or ΔgogA/ΔgtgA/ΔpipA triple mutant strains at MOI = 5. The number of CFU was enumerated 30 minutes and 8 hs after infection. Values are the fold change after 8 hs of infection (relative to the values at 30 minutes after infection) and represent the mean ± standard deviation of three independent experiments. Differences between the values of wild type and the different mutants were not statistically significant (p > 0.05). (TIF) [file ppat.1005484.s007.tif]

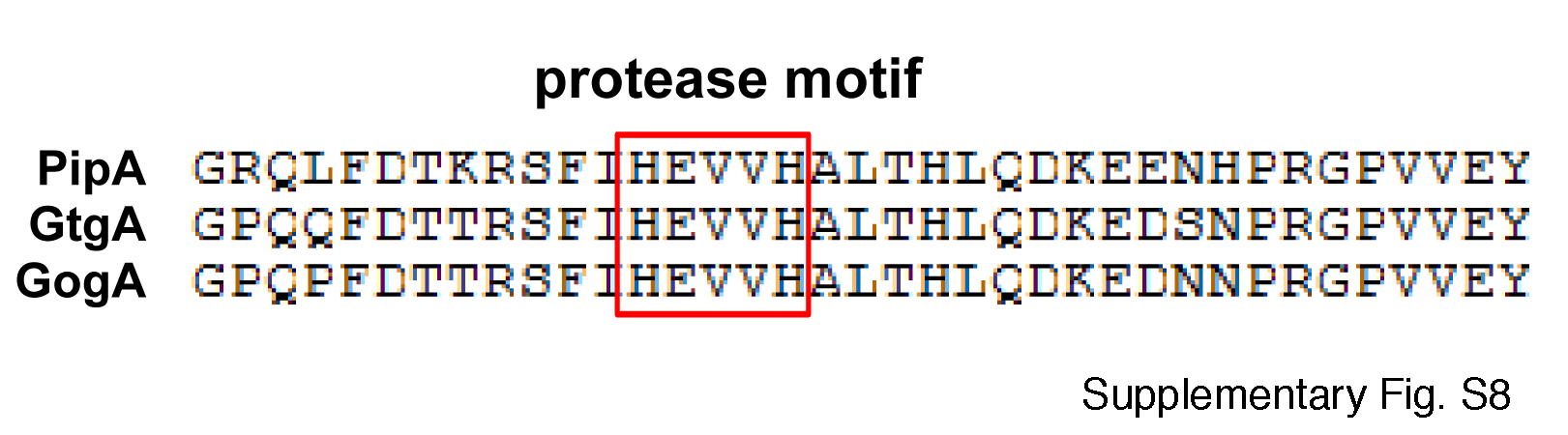

Supplement: S8 Fig — Amino acid sequence alignment of the S. Typhimurium PipA family of effector proteins depicting the location of a conserved metalloprotease Zn-binding motif. (TIF) [file ppat.1005484.s008.tif]
