# Supplementary material for: A Family of Salmonella Type III Secretion Effector Proteins Selectively Targets the NF-κB Signaling Pathway to Preserve Host Homeostasis
Source: PLoS Pathog. 2016 Mar 2;12(3):e1005484. doi: 10.1371/journal.ppat.1005484 (PMC4775039; doi:10.1371/journal.ppat.1005484)
Supplement: S1 Table — (PDF) [file ppat.1005484.s009.pdf]

<u>Table 1:</u> RelA amino terminal sequence after digestion with PipA, GogA or GtgA

	**PipA**		**GogA**		**GtgA**	
Cycle #	Seq	pm	Seq	pm	Seq 3	pm
1	Arg	9.3	Arg	7.6	Arg	11
2	Ser	5.0	Ser	3.0	Ser	5.3
3	Ala	7.5	Ala	4.6	Ala	7.3
4	Gly	4.3	Gly	2.6	Gly	4.6
5	Ser	2.5	Ser	1.4	Ser	2.2
