# Supplementary material for: A Family of Salmonella Type III Secretion Effector Proteins Selectively Targets the NF-κB Signaling Pathway to Preserve Host Homeostasis
Source: PLoS Pathog. 2016 Mar 2;12(3):e1005484. doi: 10.1371/journal.ppat.1005484 (PMC4775039; doi:10.1371/journal.ppat.1005484)
Supplement: S2 Table — (PDF) [file ppat.1005484.s010.pdf]

Table 2: Bacterial strains and plasmids used in this study

| Strain | Relevant Genotype | Source/Reference |
|---|---|---|
| | | |
| SB300 | Mouse-passed *S*. Typhimurium SL1344 | (1) |
| SB3187 | Δ*pipA* | This study |
| SB2511 | Δ*gtgA* | This study |
| SB2309 | Δ*gogA* | This study |
| SB2515 | Δ*pipA* Δ*gtgA* Δ*gogA* | This study |

| Plasmid | Description | Reference |
|---|---|---|
| | | |
| pSB5246 | Gal4-Elk1 | Obtained from Dr. Feng Shao |
| pSB5247 | Gal4-luciferase | Obtained from Dr. Feng Shao |
| pSB5248 | pGl3-NF-kB | This study |
| pSB5249 | pGl3-STAT3 | Obtained from Dr. Walther Mothes |
| pSB5250 | prk5-3xFlag-GogA | This study |
| pSB5251 | prk5-3xFlag-GtgA | This study |
| pSB5252 | prk5-3xFlag-PipA | This study |
| pSB5253 | prk5-3xFlag-PipA-E181A | This study |
| pSB5254 | pCMA-3xHA-TRIF | This study |
| pSB5255 | pCMA-3xHA-TRAF2 | This study |
| pSB5256 | pCMA-3xHA-RIP1 | This study |
| pSB5257 | pCMA-3xHA-IKKa | This study |
| pSB5258 | pCMA-3xHA-RelA | This study |
| pSB5259 | prk5-M45-STAT3 | This study |
| pSB5260 | prk5-M45-c-Jun | This study |
| pSB5261 | prk5-M45-p105 | This study |
| pSB5262 | prk5-M45-p100 | This study |
| pSB5263 | prk5-M45-mRelB | This study |
| pSB5264 | pET15b-His-GogA | This study |
| pSB5265 | pET15b-His-GtgA | This study |
| pSB5266 | pET15b-His-PipA | This study |
| pSB5267 | pET15b-His-PipA-E181A | This study |
| pSB5269 | pET15b-His-RelA(1-210aa) | This study |
| pSB5273 | pBAD24-PipA-3xFlag | This study |
| pSB5274 | pBAD24-GtgA-3xFlag | This study |
| pSB5275 | pBAD24-GogA-3xFlag | This study |

(1) Hoiseth, S.K., and Stocker, B.A. (1981). Aromatic-dependent *Salmonella typhimurium* are non-virulent and effective as live vaccines. Nature *291*, 238-239.
